# Do you feel like (A)I feel?

**DOI:** 10.3389/fpsyg.2024.1347890

**Published:** 2024-05-30

**Authors:** Alexander Tagesson, Jakob Stenseke

**Affiliations:** Department of Philosophy, Lund University, Lund, Sweden

**Keywords:** empathy, emotion, artificial intelligence (AI), perspective-taking, experience sharing

Most of us are familiar with the uncomfortable feeling that results from skepticism about others' capacity to see the world as we do. If we dig too deep into this solipsistic worry, we might feel alone in the universe—*no one can feel what it is like to be me*. One might say that it is a question of attitude. Regardless of whether the other actually *can* empathize with us, our attitude prevents us from believing it. In a correspondence article in *Nature Human Behaviour*, Perry (2023) recently made her attitude toward the prospect of empathic AI clear: they will never know how it feels to be human! This sentiment is part of a broader aversion toward the prospect of artificial empathy (AE) (e.g., Montemayor et al., 2022; Zaki, 2023). While we agree that these dystopic concerns should be taken seriously, we also believe that the debate would benefit from additional nuance. More precisely, we argue that the AI systems of today—exemplified by AE skeptics such as Perry—are not the appropriate metric to evaluate the potential for AE and should not be used as support for why people might dismiss AE as non-genuine empathy.

At the core of Perry's critique is the observation that AE is well-received until recipients realize it was generated by an AI. Perry provides two explanations for this “artificial-empathy paradox”. Firstly, “AI can learn to say the right words—but knowing that AI generated them demolishes any potential for sensing that one's pain or joy is genuinely being shared”. Secondly, human empathy is valued *because* it is demanding and finite, and since “AI entails no emotional or time cost”, it fails to indicate that “the recipient holds any unique importance”. However, we argue that neither explanation succeeds in discrediting the prospect of artificial empathy.

Empathy is a notoriously convoluted concept (see Cuff et al., 2016 for a review) and researchers often highlight cognitive-, affective-, and motivational components of empathy (Zaki, 2014; Perry, 2023). Cognitive empathy, sometimes called perspective-taking or mentalizing, is the intellectual ability to understand how the other perceives and experiences their situation (Decety and Cowell, 2014; Zaki and Ochsner, 2016; Marsh, 2018). Cognitive empathy is to a degree already achievable for AI, which can detect and identify human emotions (Montemayor et al., 2022; Perry, 2023). Affective empathy, or experience sharing, refers to how one vicariously feels and experiences the other's emotional states (Decety and Cowell, 2014; Zaki and Ochsner, 2016; Marsh, 2018). This kind of experience-sharing is potentially not obtainable for AI. Lacking lived subjective experience (Montemayor et al., 2022; Perry, 2023), trying to share the experience of an emotional human may not resonate adequately as the AI reasonably does not feel anything (Turkle, 2007). The motivational component, also called empathic concern, can be understood as a motivation to support others' wellbeing or help them alleviate suffering (Decety and Cowell, 2014; Zaki and Ochsner, 2016; Marsh, 2018). However, while it is reasonable to contest the extent to which AI can manifest affective empathy and empathic concern, we disagree with the argument that it comes down to the human recipients' attitude toward AE (Montemayor et al., 2022; Perry, 2023).

Whether empathy is valuable is *not* (solely) a question of the recipient's attitude toward the empathizer. The value of the empathy a parent directs toward her child cannot easily be discarded based on whether the child takes the parent to be a genuine empathizer or not. Similarly, the fact that some foster negative attitudes toward psychologists, which prevents them from seeking therapy that would be beneficial for them, does not discredit the value of therapy. If we view empathy as a question of attitude, then we are exposed to the solipsistic worry: what prevents us from having the “wrong” attitude toward genuine empathizers? Perry merely assumes the anthropocentric standard view: only human activities are valuable for humans (Singer, 1975). This view is also prevalent in our attitudes toward animals. Studies show that people's tendency to not attribute mental capabilities (e.g., empathy) to animals fails to live up to their own self-reported normative standards (Leach et al., 2023). This effect was attenuated when ascribing a mind and mental capabilities to humans, displaying another example of anthropocentrism. Fortunately, attitudes can change, and if it is our attitudes toward animals and AIs that are decisive for granting them genuine empathy, couldn't they also change? In another recent study, researchers manipulated participants' attitudes toward AI, making them believe that the algorithm either had a manipulative motive, a caring motive, or no particular motive. This considerably changed how participants perceived and interacted with the AI, where participants in the care motive-, or empathic concern-, group perceived it as more empathetic than participants in the two other groups. Notably, the effect was stronger for more sophisticated AI (Pataranutaporn et al., 2023). These results suggest that our attitudes and the technological sophistication of the algorithm had an impact on participants' perception of AI as being empathetic. Consequently, we believe that our malleable attitudes toward AI, combined with increased technological sophistication, will impact our perception of AE, making it more likely to be perceived as genuine empathy. Furthermore, we are also not convinced by Perry's claim that because human empathy is demanding and limited, choosing to empathize communicates the importance of the recipient to the empathizer. A recent study showed that when people learned that empathy is an unlimited resource, they became more empathic, with real-life consequences, e.g., more likely to hug an out-group member (Hasson et al., 2022). These recipients of empathy and hugs would likely not deem the empathy they received as less genuine if they learned that the empathizer believed empathy to be unlimited and not finite, which led them to feel and behave more empathically.

AI already displays cognitive empathic abilities and is capable of generating generic empathic responses. Due to vast advancements in computational approaches to emotional inference, AI has demonstrated the ability to identify human emotions (Ong et al., 2019), manifest facial emotions (Mishra et al., 2023), and to successfully facilitate empathetic interactions (Ayers et al., 2023; Sharma et al., 2023). For instance, a recent example showed how human-AI collaboration produced more empathic conversations in peer-to-peer mental health support in comparison to human-only responses (Sharma et al., 2023). In another case, healthcare professionals found AI's responses to medical questions to be almost ten times as empathetic as the responses from human physicians (Ayers et al., 2023). These generic applications of empathic displays are impressive, but when recipients learn that the empathy they received is AI-generated, they will likely not perceive it as genuine empathy. As Perry points out, “AI empathy fails to convey authentic care or to indicate that the recipient holds any unique importance”. This might not be surprising as these examples merely attest to AI's capability of emotion detection and empathic signaling and are designed to respond empathetically to anyone repeatedly. In contrast, the motivated nature of human empathy often leads people to empathize in discriminating ways (Zaki, 2014; Bloom, 2016) and with few people (Cameron and Payne, 2011; Cameron et al., 2022). Thus, generic AI applications not only deprive recipients of the feeling of uniqueness; their empathic displays are also dissimilar to how humans empathize, making them seem non-genuine. The real test of artificial empathy—by Perry's own standards—would be through personalized algorithms tuned to their human. We can envision algorithms that respond with enthusiasm and authentic care to their particular person, making *that* person more important than any other. As the relationships deepen, the personalized algorithm will be able to display more empathy toward its person, paralleling how empathy is extended in humans (Depow et al., 2021). Recipients of this kind of AE will likely perceive the care as authentic and the concern as genuine, as there are already examples of people developing strong feelings for their AI companions (Pentina et al., 2023).

While we take issue with Perry's view, we also acknowledge that much more needs to be said about the potential value and risks of AE. For instance, several ethical issues need to be seriously addressed for the responsible development and deployment of AE, e.g., regarding privacy (Lutz et al., 2019), deception (Park et al., 2023), and negative impacts on human-human relationships (Turkle, 2010). A lot more can also be said about the potential benefits of AE; e.g., how it may alleviate many of the problems associated with human empathy, such as biases, e.g., ingroup empathy bias (Cikara et al., 2014) or compassion fade (Västfjäll et al., 2014). It could potentially also lessen some of the taxing costs associated with empathy (Cameron et al., 2019) and reduce compassion fatigue (Cocker and Joss, 2016) on the part of human empathizers. To this end, researchers have called for strategic regulations of different components of empathy, i.e., affective-, cognitive- and motivational-, to attain critical outcomes (Weisz and Cikara, 2021). Similarly, we could strategically apply different components of AE to enhance human flourishing. While all these considerations must be weighed against each other to determine the overall risk-benefit of AE, contrary to what Perry claims, human attitudes toward AI do not seem to present an insurmountable obstacle.

In sum, Perry's criticism of the value of artificial empathy seems unjustified. It might turn out to be the case that full-fledged AE is unobtainable due to principled obstacles for AI to simulate human empathy. Those are other, more legitimate, reasons for not granting AI empathy than due to recipients' changeable attitudes about AE. Before making any conclusive remarks about AE being perceived as genuine empathy or not, we need to create and evaluate AI applications that better emulate human empathy. It is possible that these applications may change our attitudes so that we start perceiving artificial empathy, or parts of it, as genuine empathy.

## Author contributions

AT: Writing – original draft, Writing – review & editing. JS: Writing – original draft, Writing – review & editing.
